# Quantum Dynamical Interpretation of the Mean Strategy

**DOI:** 10.3390/e26090719

**Published:** 2024-08-23

**Authors:** Fang Wang, Peng Wang, Yuwei Jiao

**Affiliations:** 1 Chengdu Institute of Computer Application, Chinese Academy of Sciences, Chengdu 610213, China; wangfang21@mails.ucas.ac.cn; 2 School of Computer Science and Engineering, Southwest Minzu University, Chengdu 610041, China; 3 University of Chinese Academy of Sciences, Beijing 100049, China; 4 Sichuan Digital Transportation Technology Co., Ltd., Chengdu 610095, China; jiaoyuwei@scsz.com

**Keywords:** swarm intelligence, quantum dynamics, quantum harmonic oscillator model, mean strategy, multi-scale, wave function, ground state

## Abstract

The method of quantum dynamics is employed to investigate the mean strategy in the swarm intelligence algorithm. The physical significance of the population mean point is explained as the location where the optimal solution with the highest likelihood can be found once a quantum system has reached a ground state. Through the use of the double well function and the CEC2013 test suite, controlled experiments are conducted to perform a comprehensive performance analysis of the mean strategy. The empirical results indicate that implementing the mean strategy not only enhances solution diversity but also yields accurate, efficient, stable, and effective outcomes for finding the optimal solution.

## 1. Introduction

The mean strategy is a common operation in Swarm Intelligence (SI) algorithms. Many well-known algorithms such as Particle Swarm Optimization (PSO) [1], Quantum-behaved Particle Swarm Optimization (QPSO) [2], Cuckoo Search (CS) [3], and others incorporate this operation. The advantages of employing the mean strategy are manifold: firstly, it is easy to implement and parallelize; secondly, it helps avoid being trapped in local optima; thirdly, it accelerates the convergence speed of the algorithm; and fourthly, it enables a better balance between global and local search in the algorithm, leading to improved performance.

However, despite its widespread use, a precise theoretical explanation for why the mean strategy is effective is currently lacking. This knowledge gap presents a significant challenge for SI algorithms. A multitude of optimization algorithm models, particularly those based on natural systems, offer explanations for the functioning of optimization algorithms from their own perspectives, often without a complete mathematical framework [4]. The absence of a rigorous and comprehensive mathematical foundation has hindered the transformation of optimization algorithms into a formal scientific discipline [5]. The verification of the effectiveness and high efficiency of computational intelligence algorithms through numerical experimental methods and specific application means remains an important method for studying computational intelligence algorithms [6].

Fortunately, Feynman has provided us with clear guidance. He stated, “Nature is not classical, and if you want to simulate nature, you’d better turn it into quantum mechanics”. During our team’s research on the SI algorithm with quantum dynamics, we discovered that when the objective function of the optimization problem is interpreted as the potential energy within a quantum system, the optimization problem can be reformulated as the task of determining the ground state of a constrained quantum system. Consequently, it becomes feasible to develop and analyze SI algorithms by simulating the dynamic evolution process of the physical optimization system toward its ground state, thus establishing a theoretical model for SI algorithms. Table 1 shows the similarities between the quantum dynamics and the SI algorithm.

Additionally, we investigate the mean strategy in the SI algorithm using a quantum dynamic frame (QDF). This article presents several significant contributions, which are summarized as follows:

(1) Utilizing a novel theoretical method, quantum dynamics, to study and analyze the mean strategy of the optimization algorithm, offering a fresh perspective for optimization algorithm research.
(2) Employing the double well function and the CEC2013 test suite, conducting controlled experiments, performing comprehensive analyses of the mean strategy, and demonstrating its effectiveness.
(3) Analyzing the convergence process of the optimization algorithm using the wave function.

Subsequent sections delve into related work on the mean strategy of the SI algorithm and introduce the optimization problem from the viewpoint of quantum dynamics in Section 3. The theoretical underpinning of the mean strategy is expounded upon in Section 4, while Section 5 presents the experimental design and results. Conclusions and future research directions are outlined in Section 6.

## 2. Related Work

This section summarizes the related work about mean strategy on the SI algorithm.

In 2000, Kennedy demonstrated the benefits of individual particle learning from the center of the group it was currently located in, which significantly improved the algorithm’s performance [7]. In 2013, Xu utilized average velocity information in PSO to devise a feedback control strategy. By introducing average velocity into the particle position update formula, the convergence speed and accuracy of PSO were significantly enhanced [8]. During the same year, Beheshti et al. introduced Median-oriented Particle Swarm Optimization (MPSO) to conduct a global search over the entire search space, accelerating convergence speed while avoiding local optima [9]. This was achieved by incorporating the median position of particles and the worst and median fitness values of the swarm into the standard PSO. The results demonstrated that MPSO substantially improved the performance of the PSO paradigm in terms of convergence speed and the ability to find global or good near-global optima. In 2016, Yu et al. discovered that introducing the mean coordinate vector in the update formula or adding the individual average velocity term effectively enhanced the algorithm’s performance [10].

In 2004, Sun et al. discovered that in the revised QPSO, the evaluation of the parameter *L* depended on the mean best position, which demonstrated relative stability as the population evolved [11]. In 2008, Cai et al. introduced a weighting strategy for the average position of particles. This strategy involved weighting the average optimal position based on the fitness value of particles, aiming to achieve a better balance between global search and local search within the Quantum-behaved Particle Swarm Optimization (QPSO) and consequently improve its performance [12,13]. Furthermore, a QPSO algorithm based on a truncated mean stabilization strategy has been proposed, which aims to enhance population diversification and increase convergence efficiency. Experimental results have demonstrated that the search accuracy and convergence of the QPSO algorithm with the truncated mean stabilization strategy outperform those of other typical PSO variants [14].

Many other algorithms, apart from PSO and QPSO, also incorporate the mean strategy. At the 2015 International Computing and Business Intelligence Conference, Wang et al. proposed the Elephant Herding Optimization algorithm (EHO) [15], in which the elephant migration behavior with the best fitness was entirely influenced by the mean vector of elephants. In 2017, Cheung et al. enhanced the CS algorithm [16] by integrating a quantum model into the CS algorithm and introducing a non-homogeneous search strategy based on the quantum mechanism. Under this strategy, there was a probability that the cuckoo’s coordinate updating would align with the mean coordinate of birds, resulting in a significantly improved algorithm compared to the original CS algorithm. During the same year, the MGWO algorithm was developed by modifying the position update (encircling behavior) equations of the Gray Wolf Optimizer (GWO) [17], and the results illustrate that the performance of the modified variant is capable of achieving the best solutions with a high level of accuracy in classification and improved avoidance of local optima [18]. The accuracy and convergence performances of the modified variant were tested on several well-known classical sine datasets and cantilever beam design functions [19]. In 2019, Huang et al. improved the bat algorithm and introduced the Gaussian Quantum Bat Algorithm (GQBA) with quantum behavior. This algorithm utilized an average position direction flight strategy to assist bats in escaping local optimal solutions, thereby enhancing the optimization capability of the algorithm [20]. Also in 2019, Hiba et al. proposed a center-based mutation for the SHADE algorithm (CSHADE). In its mutation scheme, the base vector for SHADE’s mutation was replaced with center-based sampled candidate solutions using the normal distribution. Experimental results showed that CSHADE outperformed the Success-History Based Parameter Adaptation Differential Evolution (SHADE) algorithm across a majority of benchmark functions in terms of solution accuracy [21].

The various algorithms and their enhanced versions mentioned above appear to integrate a fundamental operation to ensure the optimization algorithm’s performance—namely, the utilization of mean information. The effective utilization of mean information can enhance the solution accuracy of algorithms. However, to our knowledge, these algorithms have not provided a clear explanation of the specific significance of mean information in the SI algorithm.

In 2019, our team’s Ye et al. proposed a new multi-scale Quantum Harmonic Optimization Algorithm (MQHOA) with a Truncated Mean Stabilization (TS-MQHOA) policy. Theoretical and experimental analyses indicate that the truncated mean stabilization strategy helps diversify populations and improve convergence efficiency. Furthermore, experimental results on complex test problems demonstrate that the proposed TS-MQHOA, in most function evaluations, achieves better convergence toward the global optimum compared with several renowned heuristic algorithms based on swarm intelligence [22]. While truncating the mean has some effect, it leads to a loss of group diversity, and we believe that the authors’ theory and experimental methods are incomplete. Subsequently, in the same year, the mean strategy of the MQHOA algorithm is explained from the perspective of local optimal solutions, and various replacement strategies are compared. Experimental results show that the algorithm’s performance is not significantly improved by the random migration strategy [23]. Then, in 2024, we conducted a parameter estimation of the ground state wave function through the maximum likelihood estimation method to explain the mathematical significance of the population mean point from the dynamics perspective [24]. Building on this work, we now aim to elucidate the essential significance of the mean strategy from the perspective of quantum dynamics and demonstrate the effectiveness of the mean strategy using a quantum harmonic oscillator model.

## 3. Quantum Dynamic Frame for Swarm Intelligence Algorithms

**Figure 1:**
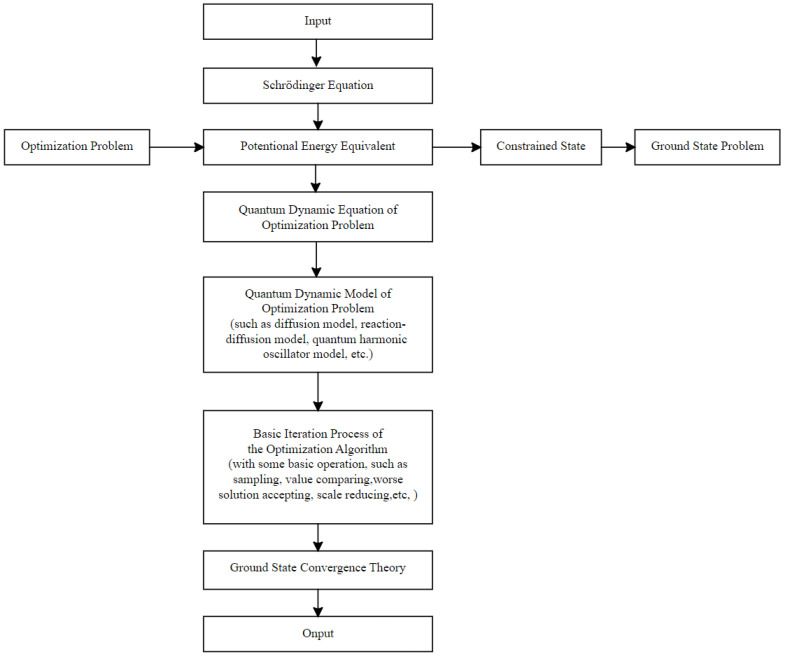
The quantum dynamic framework for the swarm intelligence algorithm.

The quantum dynamics frame (QDF) for swarm intelligence algorithm is a paradigm that transforms the iterative evolution process of optimization algorithms into the evolution process of a quantum constrained state to the ground state (Figure 1).

### 3.1. Quantum Dynamic Equation of Optimization Problem

From a physics perspective, the iterative process of optimization algorithms operating on a classical computer can be likened to dynamics evolving over time. Some scholars have begun to recognize the connection between optimization algorithms and dynamical systems [25]. To establish a mathematical–physical model of the optimization algorithm, the most direct approach is to seek a time evolution equation that describes the optimization algorithm. This equation is known as the kinetic equation. Therefore, we naturally think of the Schrödinger equation. The objective function in the optimization problem is considered as the constrained potential energy in the quantum system, thereby transforming the solution of the optimization problem into the solution of the constrained ground state wave function problem. The Schrödinger equation of a single particle moving in one dimension in quantum mechanics is as follows:

(1) $$i\hbar \frac{\partial \Psi (x,t)}{\partial t}=\left(-\frac{\hbar^{2}}{2m}\frac{\partial^{2}}{\partial x^{2}}+V\left(x\right)\right)\Psi \left(x,t\right).$$

The dynamic equation of the optimization algorithm is not directly related to the microscopic particle dynamics equation. In order to obtain the Quantum Dynamic Equation (QDE) of the optimization algorithm, the objective function f(x) in the optimization algorithm is utilized as the constraint for the quantum system’s potential energy, expressed as V(x)=f(x). By establishing this equivalence, the time-dependent Schrödinger equation is transformed into the QDE of the optimization algorithm

(2) $$i\hbar \frac{\partial \Psi (x,t)}{\partial t}=\left(-\frac{\hbar^{2}}{2m}\frac{\partial^{2}}{\partial x^{2}}+f\left(x\right)\right)\Psi \left(x,t\right).$$

Since the iterative process of the optimization algorithm is a process of continuous motion evolution, this project regards the iterative evolution process of the optimization algorithm as a dynamic process and establishes the time-dependent dynamic equation of the optimization algorithm.

### 3.2. Diffusion Model of QDF

In optimization problems, the objective function f(x) is considered as a black box, and the analytic expression of the objective function cannot be directly obtained in optimization problems. Therefore, Taylor expansion has been used to study the influence of the objective function f(x) on the dynamic process.

In mathematics, the Taylor series is the expression of a function f(x) for which the derivatives of all orders exist at a point $x_{0}$ in the domain of f(x) in the form of the power series, so let the estimate of the objective function under the black box be $f_{B}\left(x\right)$; then,

(3) $$f\left(x\right)\approx f_{B}\left(x\right)=\sum_{j=0}^{n}\frac{f^{n}\left(x_{0}\right)}{j!}\left(x-x_{0}\right).$$

Then QDE is transformed into

(4) $$i\frac{\partial \Psi (x,t)}{\partial t}=\left(-D\frac{\partial^{2}}{\partial x^{2}}+f_{B}\left(x\right)\right)\Psi \left(x,t\right).$$

Since the Taylor zero-order approximation of the objective function is constant, it may be scaled and translated to 0, and let $D=\frac{\hbar }{2m}$. Then, the dynamic equation is reduced to the free particle equation:

(5) $$i\frac{\partial \Psi (x,t)}{\partial t}=-D\frac{\partial^{2}\Psi \left(x,t\right)}{\partial x^{2}}.$$

The free particle equation in quantum mechanics is the diffusion equation of the imaginary diffusion coefficient. In order to facilitate the analysis and operation, in Equation (5), after the real time is converted into an imaginary time, Equation (6) is a standard diffusion equation:

(6) $$\frac{\partial \Psi (x,\tau )}{\partial t}=D\frac{\partial^{2}\Psi \left(x,\tau \right)}{\partial x^{2}}.$$

Its corresponding green function is a normal function

(7) $$G\left(x^{\prime },▽\tau ;x_{0},0\right)=\frac{1}{\sqrt{4\pi D\tau }}e^{-\frac{{\left(x^{\prime }-x\right)}^{2}}{4D▽\tau }}.$$

The Taylor zero-order approximation of the objective function corresponds to the black-box model of the objective function. This shows that the optimization algorithm’s population is normally sampled when the objective function is a black box. The sampling formula is

(8) $$x^{\prime }=x+\sigma N\left(0,1\right),$$

where $\sigma =\sqrt{\hbar \Delta \tau /m}$ is the scale of the sampling.

### 3.3. Reaction–Diffusion Model of QDF

The objective function is performed as a first-order Taylor expansion, and then the QDE of the optimization problem is the diffusion equation with the drift term.

(9) $$i\frac{\partial \Psi (x,t)}{\partial t}=\left(-D\frac{\partial^{2}}{\partial x^{2}}+f_{B}\left(x\right)\right)\Psi \left(x,t\right)=\left(-D\frac{\partial^{2}}{\partial x^{2}}+f^{\prime }\left(x_{0}\right)\left(x-x_{0}\right)\right)\Psi \left(x,t\right).$$

It is a diffusion-action equation of the imaginary diffusion coefficient.

Equation (9) shows that by comparing the values of the two samples before and after, the slope is obtained. Under the QDF, the values of two adjacent samples are expressed by energy $E_{a}$ and $E_{b}$ ($E_{a}$ is the low-energy state and $E_{b}$ is the high-energy state), respectively, according to the energy superposition Equation (15), and the superposition of *n* energy states in the formula is approximately the superposition of two energy states $E_{a}$ and $E_{b}$. That is the two-energy level approximation (TELA). At this point, the energy superposition formula is approximately expressed as a two-level formula:

(10) $$\Psi \left(x,t\right)=c_{a}\psi_{a}exp\left(-iE_{a}t\right)+c_{b}\psi_{b}exp\left(-iE_{b}t\right).$$

The probability of two energy levels is mainly determined by the exponential term of Equation (15), so the worse solution acceptance probability can be approximated as

(11) $$P_{b}\approx \frac{exp(-iE_{b}t)}{exp\left(-iE_{a}t\right)+exp\left(-iE_{b}t\right)}\approx e^{-(E_{b}-E_{a})it}.$$

By TELA, the algorithm introduces a priori knowledge based on the assumption of the objective problem, so as to orient the movement direction of the particles and worse solution acceptance probability.

(12) $$P_{b}=\left\{\begin{array}{cc} 1, & if\;f\left(x_{b}\right)<f\left(x_{a}\right)\\ e^{-(E_{b}-E_{a})it}, & if\;f\left(x_{b}\right)⩾f\left(x_{a}\right) \end{array}\right..$$

After the Wick rotation, the probability of worse solution acceptance is

(13) $$P_{b}=\left\{\begin{array}{cc} 1, & if\;f\left(x_{b}\right)<f\left(x_{a}\right)\\ e^{-(E_{b}-E_{a})\tau }, & if\;f\left(x_{b}\right)⩾f\left(x_{a}\right) \end{array}\right..$$

### 3.4. Quantum Harmonic Oscillator Model of QDF

If the sampling point is the extreme point, the QDE of the objective function under Taylor’s second-order approximation near the global best point is isomorphic to the quantum harmonic oscillator equation:

(14) $$i\frac{\partial \Psi (x,t)}{\partial t}=\left(-D\frac{\partial^{2}}{\partial x^{2}}+f_{B}\left(x\right)\right)\Psi \left(x,t\right)=\left(-D\frac{\partial^{2}}{\partial x^{2}}+\frac{1}{2}f^{\prime \prime }\left(x_{0}\right){\left(x-x_{0}\right)}^{2}\right)\Psi \left(x,t\right).$$

In this case, the complicated objective function is approximated by the harmonic oscillator potential function of a single peak. Then, solving the optimization problem is transformed into solving the ground state wave function of the quantum harmonic oscillator.

### 3.5. Basic Iteration Process of Optimization Algorithm

When explaining the iterative process of swarm intelligence algorithm from the perspective of quantum dynamics, the core iterative operation of these algorithms becomes a fundamental requirement within the theoretical framework of quantum dynamics. The three models derived from QDF can provide a general basic iterative process (BIP) (see Algorithm 1).
**Algorithm** **1** Pseudocode of BIP with mean strategypopulation initialization within the defined domain**while** does not meet the algorithm termination conditions **do**    **while** does not meet the ground state condition **do**        current scale group normal random sampling        adjusting particle density based on the sampling of the objective function    **end while**    scale reduction**end while**output

The BIP of this swarm intelligence algorithm can serve as the foundation for the design and improvement of the swarm intelligence algorithm.

### 3.6. Ground State Convergence Theory

The general solution of Equation (2) is

(15) $$\Psi \left(x,t\right)=\sum_{n}c_{n}\psi_{n}\left(x\right)exp\left(-\frac{iE_{n}t}{\hbar }\right).$$

In the paper, the Planck constant *ℏ* is defined as 1 in natural units. And the solution after the Wick rotation [26] is

(16) $$\Psi \left(x,\tau \right)=\sum_{n}c_{n}\psi_{n}\left(x\right)exp\left(-E_{n}\tau \right).$$

Following Equation (16), the BIP [27] inevitably converges to the optimal solution as the iterations progress [28]. The solution |Ψ(τ)〉 is approximated by the test state $|\Psi (\vec{\phi }\left(\tau \right))\rangle$, $\vec{\phi }\left(\tau \right)=\left(\phi_{1}\left(\tau \right),\phi_{2}\left(\tau \right),\ldots ,\phi_{N}\left(\tau \right)\right)$. As τ approaches infinity, the ground state is obtained by $\Psi_{arb}\left(x,\tau \right)$’s evolution.

(17) $$\left\{\begin{array}{cc} \; & \frac{dE_{\tau }}{d\tau }=-\sum_{j,k}C_{j}A_{j,k}^{-1}C_{k}\leq 0\\ \; & \lim_{\tau \to \infty }\Psi \left(x,\tau \right)=c_{0}\Psi_{0}\left(x\right) \end{array}\right.$$

where $A_{jk}=Re\left(\frac{\partial \langle \phi |}{\partial \phi_{j}}\frac{|\phi \rangle }{\partial \phi_{j}}\right),C_{j}=-Re\left(\frac{\partial \langle \phi |}{\partial \phi_{j}}H|\phi \rangle \right)$ [28]. This is the convergence theory of quantum dynamic frame (QDF).

## 4. Theory of Mean Strategy

### 4.1. Analysis of the Characteristics of the Quantum Harmonic Oscillator Model

The quantum harmonic oscillator is a well-known model in quantum mechanics, extending the classical harmonic oscillator. It is particularly valuable for approximating the small vibrations of microscopic systems near equilibrium points. Owing to its simple analytical solutions, it finds a wide application in physics for modeling the complex vibrations of crystals around their equilibrium positions.

Furthermore, in optimization problems, the objective function is often complex, but only the probability distribution near the global optimal solution is essential. Therefore, the quantum harmonic oscillator potential well model can be employed to approximate the complicated objective function. The central position of the potential well represents the mean point position, where the maximum probability of finding the global optimal solution occurs [24].

**Figure 2:**
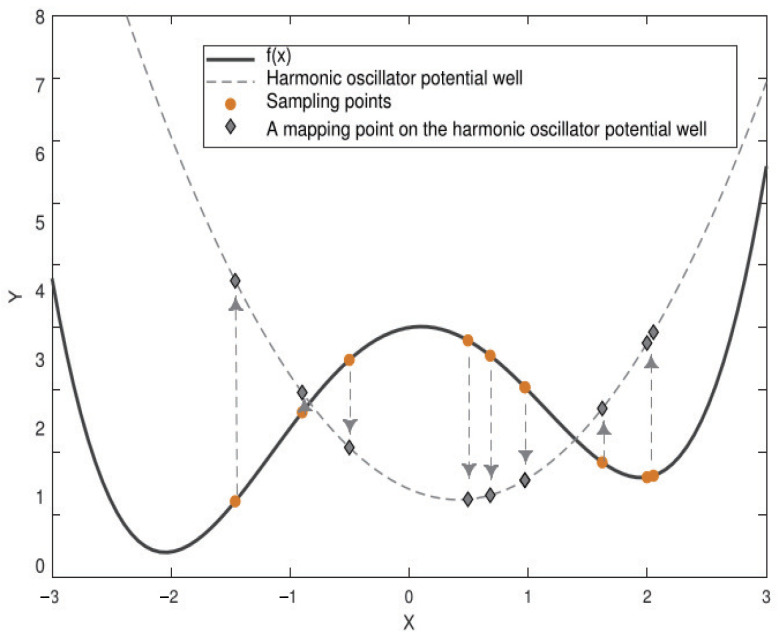
The approximation of the target function (double well function) by the two-dimensional double well harmonic oscillator potential. The dashed line represents the target function after a second-order approximation, which is the harmonic oscillator potential, while the solid line represents the target function, and the dotted arrows represent the mapping from the sampling point to the harmonic oscillator.

**Figure 3:**
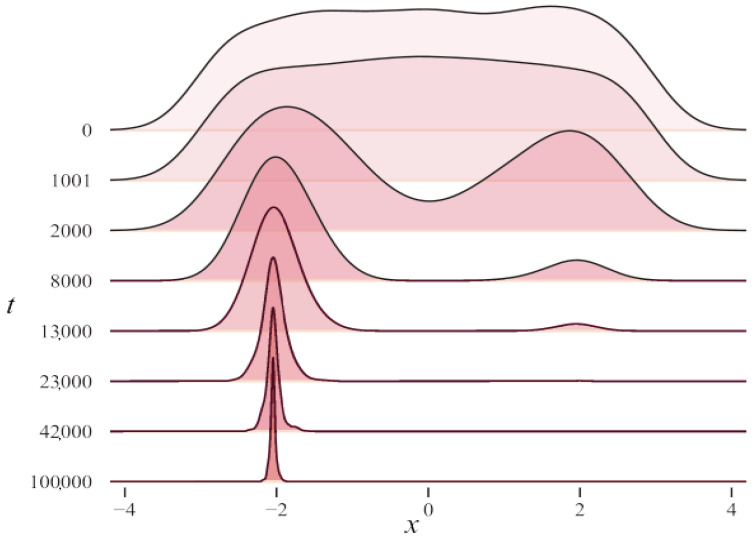
The ground state wave function evolution of the quantum harmonic oscillator.

As the image of the quantum harmonic oscillator (Figure 2) and its ground state wave function (Figure 3) are centrosymmetric, we regard the central position as the targeted solution. Placing emphasis on the center within the population leads us towards the optimal solution.

In conclusion, selecting the population’s center as the position with the highest probability of the optimal solution can enhance our comprehension of the underlying physical principles and yield improved outcomes in practical applications.

### 4.2. The Physical Implications of the Mean Strategy

The lowest energy state of the quantum harmonic oscillator is known as the ground state, and the corresponding wave function is denoted by

(18) $$\Psi \left(x\right)=\frac{1}{{\left(2\pi \sigma^{2}\right)}^{\frac{1}{4}}}exp\left(-\frac{x^{2}}{4\sigma^{2}}\right),$$

where $\sigma =\frac{1}{\sqrt{2}a}$. This expression represents the standard form of the Gaussian function. When the equivalent subsystem reaches the ground state, the probability distribution of the solution follows a normal distribution $N\left(\bar{x},\sigma^{2}\right)$, where $\bar{x}$ represents the mean of the particles.

Based on the properties of the normal distribution, if the solution error is represented by ε, the integral $\int_{x-\varepsilon }^{x+\varepsilon }N\left(\bar{x},\sigma^{2}\right)dx$ can be maximized when the sampling point $x=\bar{x}$; thus, the probability of finding the optimal solution near the mean point is maximized. In optimization problems, although the objective function is often complicated, it suffices to know the probability distribution around the global optimal solution. Therefore, we can use the quantum harmonic oscillator potential well model to approximate the objective function, and its central position (mean point position) serves as the position with the maximum probability of finding the global optimal solution.

In Figure 2, we project the actual sampling point of the target function $f\left(x\right)$ onto the approximate function of the objective function. Since the approximation function is a quadratic function with upward opening, (19) obviously holds.

(19) $$\left\{\begin{array}{cc} \; & f_{\sigma }\left(\bar{x}\right)<f_{\sigma }\left(x_{worst}\right)\\ \; & \bar{x}=\sum_{i=1}^{k}x_{i}/k\\ \; & x_{worst}=\arg\max_{x_{i}}\left\{f\left(x_{i}\right)\right\},\ldots ,k\}. \end{array}\right.$$

Here, *k* denotes the number of sampling points. The fitness $f_{\sigma }\left(x\right)$ of the sampling point mean $\bar{x}$ is always better than the worst solution in the process of approximating the objective function $f\left(x\right)$ by the quadratic function with upward opening. Therefore, we can use a simple strategy based on the mean information, i.e., generate a new solution using the coordinate mean of the population, to replace the current worst solution and complete the migration of the entire population towards the mean coordinate of the group. This is called “mean strategy”.

**Figure 4:**
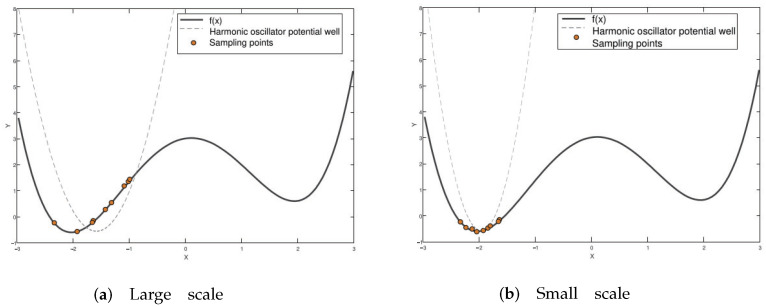
The approximation process of a two-dimensional double well potential of the harmonic oscillator for the objective function (double well function): (**a**) approximation at a large scale, (**b**) approximation at a small scale.

The complexity of the objective function makes it impossible to achieve accurate approximations using a single-scale harmonic oscillator potential energy. Therefore, a multi-scale harmonic oscillator potential well can be used to estimate the position of the minimum value of the complex function. As depicted in Figure 4a, at a larger scale, the potential well’s constraint on the harmonic oscillator is minimal, resulting in only slight initial positioning of the minimum value. Conversely, Figure 4b shows that at a smaller scale, the potential well constrains the motion of the harmonic oscillator within a limited range, allowing for precise localization of the minimum value’s position.

### 4.3. Mean Strategy of SI Algorithm

If the sampling point is the extreme point, the QDE of the objective function is the quantum harmonic oscillator equation. The corresponding pseudo code (Algorithm 2) of BIP with the mean strategy is as follows:
**Algorithm** **2** Pseudocode of BIP with mean strategy**Input:** particles number *k*, left boundary LB, right boundary RB, domain [LB,RB], scale σ=UB−LB **Output:** $\arg\min_{x}\left\{(f(x)\right\}$
Generate *k* copies of free particle in the domain**while** termination condition is not met **do**    **while** not reached ground state **do**        Normal sampling according to Equation (8)        Value comparing according to Equation (9)        Worse solution accepting according to Equation (13)    **end while**    Mean value replacing (Mean strategy) according to Equation (19)    Scale reducing**end while**


This algorithm is divided into two internal and external loops. The inner loop is used to find the ground state at the anchored scale. It begins with normal sampling, compares the current sampled value with the value from the previous sampling, retains the optimal solution, and then determines whether the ground state has been reached at the current scale. If the ground state has not been reached, the process continues as described above. If the ground state is reached, it enters the outer loop, accepts worse solutions, and performs scale reduction operations. Meanwhile, the outer loop controls the entire iteration process of the algorithm based on the requirements for solution accuracy and the number of iterations.

## 5. Experiment

The experiment aimed to investigate the impact of the mean strategy on the algorithm from two perspectives: process and results. The former analyzed the role of the mean strategy in the BIP-mean algorithm by considering the objective function (double well function) as a white box. Theoretical analysis was conducted to understand how the mean strategy affects the algorithm’s performance. The latter considered the objective function (CEC2013 test suit) as a black box and selected three algorithms from the BIP cluster to observe and analyze the impact of the mean strategy on algorithm performance. These algorithms are the BIP algorithm without using the mean strategy, the BIP-mean algorithm with the mean strategy, and the MQHOA-wmn algorithm with incomplete information but using the mean strategy.

### 5.1. Test Functions

In this article, the test function refers to the objective function selected for evaluating the algorithm.

#### 5.1.1. Double Well Function

The experiment used a test function known as the double well function (DWF), which is an important model originating from quantum physics. The DWF has been used for decades to analyze energy, wave function, and tunnel effect, making it an ideal model for optimization algorithm research [29,30]. It has also been applied to test quantum-inspired optimization algorithms and demonstrate the principle of quantum annealing for optimization problems [31,32,33].

The DWF can be accurately controlled by adjusting its function parameters [34], including the potential well depth, potential well distance, and barrier height. This allows for precise tracking of the physical image of the iterative process of the algorithm, especially in studying the dynamic characteristics of the wave function.

The one-dimensional asymmetric DWF is expressed as follows:

(20) $$f\left(x\right)=V\frac{{\left(x^{2}-a^{2}\right)}^{2}}{a^{4}}+\delta x.$$

The DWF obtains a minimum near x≈±a, and the difference between the two minima is determined by the parameter δ. The smaller δ is, the smaller the gap between the two minima. The parameter *V* determines the barrier between the minima, and the larger *V* is, the higher the barrier will be. By adjusting the three parameters *a*, δ, and *V*, the position and difficulty of the optimal solution of the objective function can be changed. Since the optimal solution of the DWF is not at the center of all extreme points, it can avoid the sampling advantage of optimization algorithms for the center position.

The high-dimensional asymmetric DWF can be realized by superimposing multiple one-dimensional DWFs. Its expression is as follows:

(21) $$f_{n}\left(x\right)=\sum_{i=1}^{n}V\frac{{\left(x_{i}^{2}-a^{2}\right)}^{2}}{a^{4}}+\delta x_{i}$$

As a standard test function, the high-dimensional asymmetric DWF can comprehensively analyze the convergence process and performance of the algorithm through the adjustment of parameters and dimensions. The number of global optimum and the number of extreme points of the high-dimensional asymmetric DWF are 1 and $2^{n}$, respectively.

Overall, the DWF is a versatile and relevant test function for optimization algorithm research due to its clear physical background and controllable parameters. Its one-dimensional and high-dimensional forms can provide valuable insights into the convergence and performance of optimization algorithms.

#### 5.1.2. CEC2013 Test Suite

Table 2 lists the CEC2013 test suite, where *n* represents the dimensionality of the problem.This test set comprises a total of 28 functions that are categorized into unimodal functions ($f_{1}∼f_{5}$), multimodal functions ($f_{6}∼f_{20}$), and composite functions ($f_{21}∼f_{28}$) based on their functional structures. The domain of definition for all functions is $\left[-100,100\right]$.

### 5.2. Parameter Settings

To examine the effectiveness of the mean strategy, this study selected three representative BIP-like algorithms for experimental observation: BIP, BIP-mean (BIP with mean strategy), and the worst particle replaced by the mean position without the best and the worst (MQHOA-wmn). While BIP is a bare-bones algorithm without a mean strategy, BIP-mean has only one additional mean strategy than BIP. On the other hand, MQHOA-wmn is a BIP-like algorithm with a mean strategy but incomplete mean information that eliminates the optimal and worst solutions. To ensure consistency, we set the number of sampling points (*k*) in the experimental group to 30, while the scale reduction coefficient (also known as scale coefficient) was set to 2. All control experiments were repeated 51 times, and the maximum number of iterations for a single operation was 10,000 ×d, where *d* represented dimensionality. For the experimental dimension, we set it to 30 (Table 3).

### 5.3. Convergence Analysis

**Figure 5:**
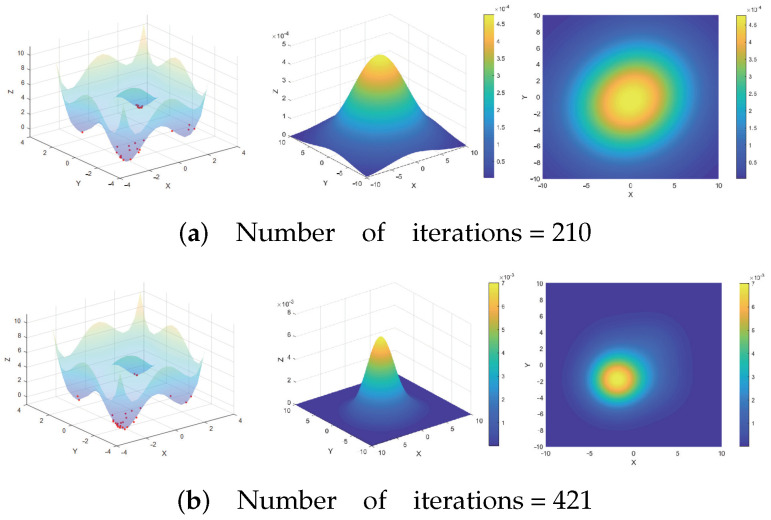

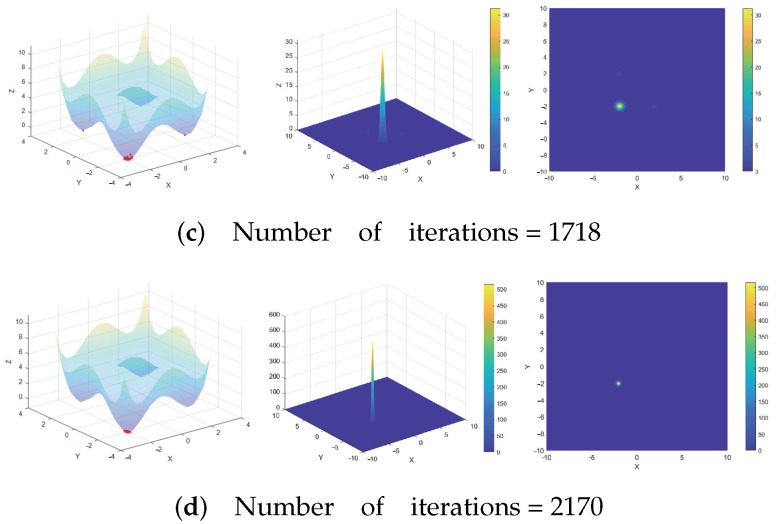
Convergence process of wave function and the red dots represent sampling points.

Through the analysis of the wave function evolution process, this paper investigates the convergence of the algorithm. Figure 5 displays a two-dimensional schematic diagram, particle distribution state, and top view of the ground state wave function on the objective function from left to right, with *t* representing the number of iterations. The results indicate that as the scale gradually decreases, the wave function converges towards the global optimal region, and the algorithm transitions from a global search to a local search.

### 5.4. Exploration and Exploitation

Exploration and exploitation are two abilities of SI algorithms. The process of accelerating convergence is also a conversion between exploration and exploitation. The mean-oriented learning strategy based on multi-scale ground state wave functions effectively promotes the transformation of sampling populations to the exploitation process. Exploration can be understood as the process of completely searching for new areas in the solution space, while exploitation can be understood as the process of searching for solutions near previously visited points [35]. Reasonably adjusting the relationship between exploration and exploitation is beneficial to improving the performance of the algorithm. Ref. [36] indicates that the key to the optimization process is to balance exploration and exploitation. However, the two are not only opposed to each other but are, sometimes, mutually reinforcing, and promoting exploitation does not necessarily harm exploration. In BIP, the iterative process of particles is affected by the scale σ. In the early iteration stage, the scale σ is large. When the system reaches the ground state, the ground state wave function at this scale is widely distributed, and many particles are in previously unknown regions and in the exploration process. As the scale decreases, the ground state wave function gradually converges, and a large number of particles turn from global to optimal solutions. The transformation between the exploration process and the exploitation process is analyzed by tracking the iterative process of particles and the convergence process of the wave function.

Figure 5 illustrates that in the early stages of iteration, particles are widely dispersed and many sampling points are in the exploration state due to the large search space. As the algorithm progresses and the scale reduces, particles gradually converge towards the global optimal solution, transitioning into an exploitation state. Concurrently, the ground state wave function also converges towards the global optimal solution, improving the certainty and enabling a more precise search. The mean strategy facilitates this process by promoting exploitation.

**Figure 6:**
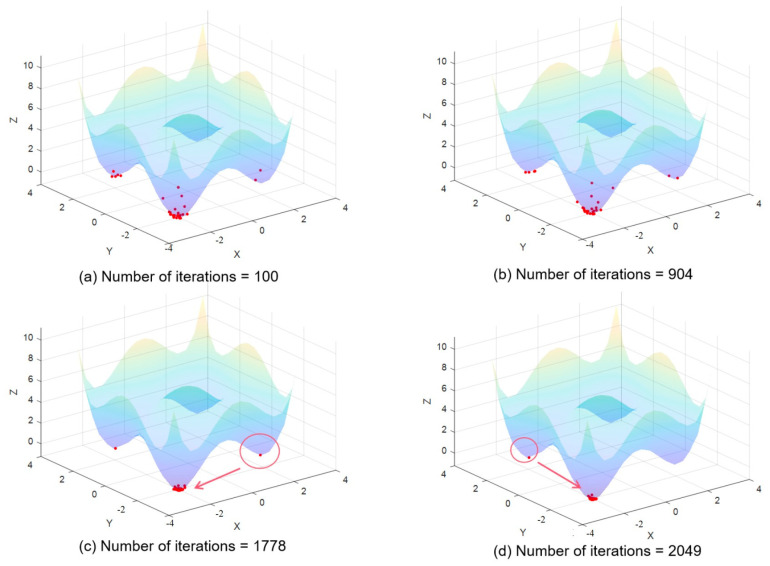
The promotion of mean direction learning strategy for exploitation process and the red dots represent sampling points.

Figure 6 demonstrates that the mean strategy aids in improving the evaluation and solving the accuracy of the algorithm. Sampling points with poor fitness have a higher likelihood of entering the exploitation state by learning towards the mean value. This method also promotes convergence by helping sampling points transition from exploration to exploitation, thereby enhancing the algorithm’s performance. Furthermore, mean-direction learning is effective in escaping local optima, which otherwise present a challenge with traditional sampling methods.

### 5.5. Solution Accuracy Discussion of Mean Strategy

To validate the effectiveness of the mean strategy, we conducted a control experiment. The results presented in Table 4 demonstrate that BIP-mean outperforms both BIP and MQHOA-wmn in terms of solving error, solving error mean, and optimal solution. Although MQHOA-wmn also exhibits outstanding performance [23], its lack of optimal and worst solutions in calculating the mean coordinates results in a larger evaluation error of the optimal solution under the current ground state, compared to BIP-mean. Additionally, due to a shortage of samples in the maximum likelihood estimation process, MQHOA-wmn may experience a deficiency in this stage when compared to BIP-mean.

### 5.6. Stability Discussion of the Mean Strategy

**Figure 7:**
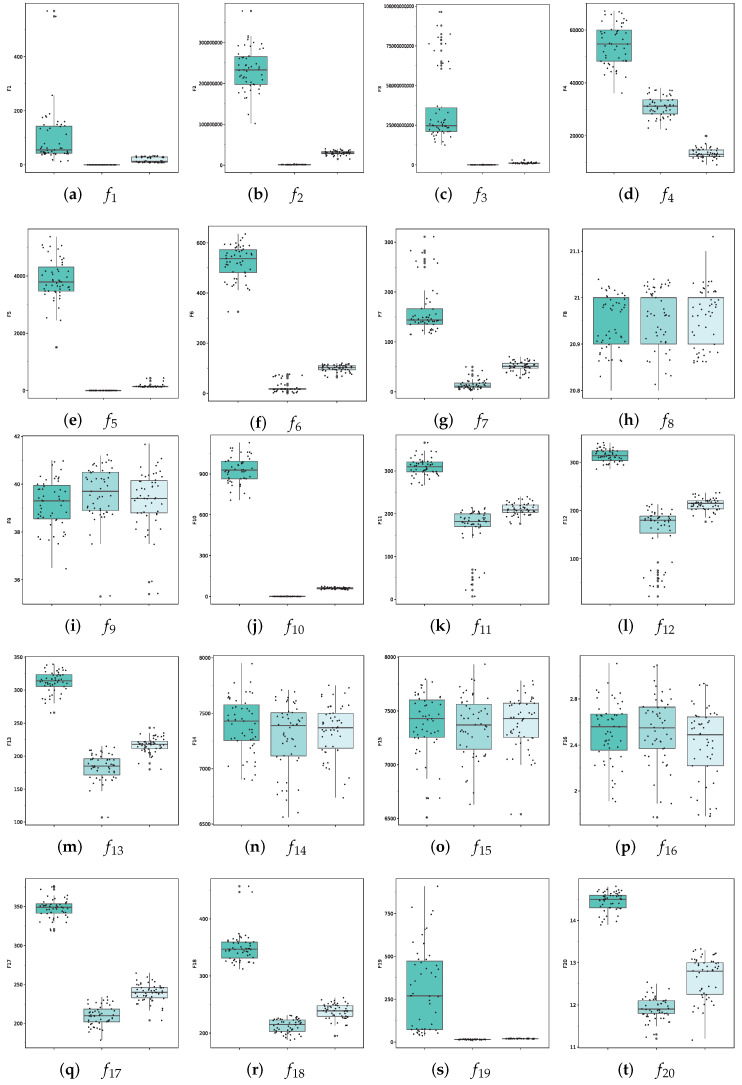

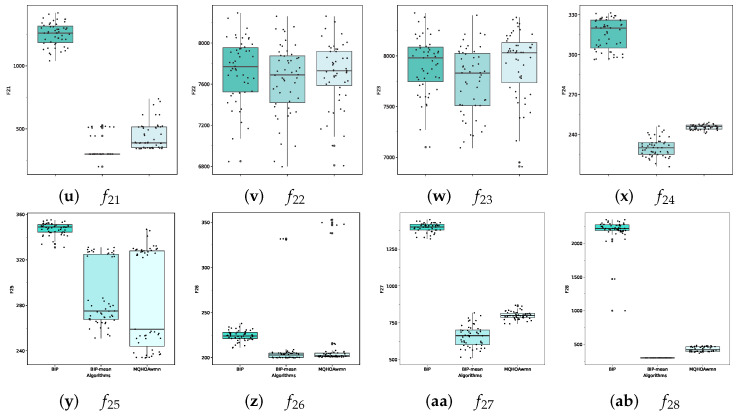
Boxplots of algorithms on CEC2013; the experiment dimension was 30, it was repeated 51 times, and a horizontal black line is the confidence interval of the median.

To capture the characteristics of solving error distribution, we created boxplots diagrams based on data from each experiment of the algorithm (Figure 7). These diagrams display the maximum, minimum, median, and upper and lower quartiles of the algorithm’s optimization results in repeated experiments, allowing us to directly evaluate the stability of the algorithm’s solution. When optimizing the algorithm, a flatter boxplots diagram with an ordinate tending towards zero indicates higher stability.

Our boxplots diagrams clearly demonstrate that the mean strategy experimental group, which utilized complete population coordinate information, achieved superior accuracy and stability in the CEC2013 test. This confirms the stability of the mean strategy in quantum systems with objective function potential energy constraints.

### 5.7. Effectiveness Discussion of the Mean Strategy

To assess the effectiveness of the mean strategy on the optimization algorithm, we conducted a Wilcoxon signed rank test on the results obtained from experiments conducted on the control group. If the mean value of repetitions of the algorithm is reduced upon adding the mean strategy behavior, as compared to the control group without this behavior, and if the Wilcoxon signed rank test yields a true result (significance level less than 0.05, *p*-Value ≪0.05), then we consider the results of the algorithm with a mean strategy behavior to be significantly better than those of the original algorithm, and vice versa.

The experimental results are presented in Table 5. Our findings show that the optimization results achieved by BIP-mean on 23 test functions are significantly better than those of the original BIP algorithm in the series control experiments. While the mean strategy adopted by MQHOA-wmn is based on fewer samples, it still exhibits significant advantages over BIP on 21 test functions. Moreover, BIP-mean outperforms MQHOA-wmn on 19 test functions. These results demonstrate the effectiveness of mean replacement and suggest that a greater sample size leads to a more accurate estimation of the optimal solution.

### 5.8. Solving Speed Discussion of Mean Strategy

**Figure 8:**
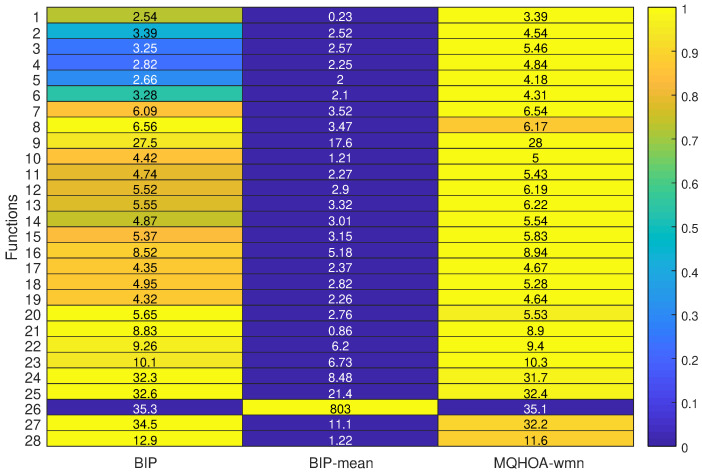
The average time taken by the algorithm for the corresponding test function, measured in seconds (s); the legend on the right is the relative time after taking the logarithm; the closer it is to 0, the faster the algorithm is.

To visualize the average iteration time of our experiments, we calculated and plotted the data in Figure 8. To provide a clear representation of the results, we normalized the time data and applied color coding. The darker areas indicate that less time was consumed during the execution of the algorithm. Our findings suggest that BIP-mean outperforms BIP and MQHOA-wmn in terms of time efficiency. Specifically, BIP-Mean exhibits a significant advantage over the other two algorithms in minimizing time consumption across all test functions studied.

## 6. Conclusions and Future Works

In this paper, the physical significance of the mean strategy in the SI algorithm is revealed and explained by the quantum harmonic oscillator model in quantum dynamics. We prove that the highest probability of obtaining the optimal solution occurs when the mean value of the population coincides with the lowest potential energy point, subject to the current constraint. This approach strikes an excellent balance between exploration and exploitation by increasing population diversity at the onset of the iteration and promoting faster convergence during the middle and later stages of the iteration. Our experimental results demonstrate that employing the mean strategy yields superior accuracy, speed, stability, and efficiency in finding the optimal solution. Future research should focus on developing more accurate approximations of the objective function under complex potential well constraints.

## Figures and Tables

**Table 1:** The basic correspondence between the SI algorithm and quantum dynamics.

SI Algorithm	Quantum Mechanics
Objective Function	Potential Field
Solution to Optimization Problem	Wave function
Optimal Solution	Ground State Wave Function
Local Optimal Solution	Excited State Wave Function
Dynamic Equation of SI Algorithm	Schrödinger Equation
Iteration Process	Evolution of Wave Function
Probability Distribution of the Solution	Modulus Square of the Wave Function
Reception for Worse Solution	Two Energy Level Approximation
Population Sampling	Kinetic Energy

**Table 2:** Test functions of CEC 2013 single objective optimization benchmark suite.

Categories	No.	Functions
Unimodal Functions	1	Sphere Function
	2	Rotated High Conditioned Elliptic Function
	3	Rotated Bent Cigar Function
	4	Rotated Discus Function
	5	Different Powers Function
Basic Multimodal Functions	6	Rotated Rosenbrocks Function
	7	Rotated Schaffers F7 Function
	8	Rotated Ackleys Function
	9	Rotated Weierstrass Function
	10	Rotated Griewanks Function
	11	Rastrigins Function
	12	Rotated Rastrigins Function
	13	Non-Continuous Rotated Rastrigins Function
	14	Schwefel’s Function
	15	Rotated Schwefel’s Function
	16	Rotated Katsuura Function
	17	Lunacek Bi_Rastrigin Function
	18	Rotated Lunacek Bi_Rastrigin Function
	19	Expanded Griewanks plus Rosenbrocks Function
	20	Expanded Scaffers F6 Function
Composition Functions	21	Composition Function 1 (Rotated)
	22	Composition Function 2 (Unrotated)
	23	Composition Function 3 (Rotated)
	24	Composition Function 4 (Rotated)
	25	Composition Function 5 (Rotated)
	26	Composition Function 6 (Rotated)
	27	Composition Function 7 (Rotated)
	28	Composition Function 8 (Rotated)

**Table 3:** Parameter setting.

Parameter	*k*	σ	Repeat Times	Max Iterations
setting	30	2	51	300,000

**Table 4:** The average error, standard deviation, and minimum error of each algorithm, the bold part is the optimal solution.

f(x)	BIP			BIP-Mean			MQHOA-wmn		
	**MEAN-ERR**	**STD-ERR**	**MIN-ERR**	**MEAN-ERR**	**STD-ERR**	**MIN-ERR**	**MEAN-ERR**	**STD-ERR**	**MIN-ERR**
f1	1.02 × 10^2^	1.09 × 10^2^	1.24 × 10^1^	**3.34 × 10^−13^**	**1.15 × 10^−13^**	**2.27 × 10^−13^**	2.19 × 10^1^	1.03 × 10^1^	6.64 × 10^0^
f2	2.34 × 10^8^	5.19 × 10^7^	1.02 × 10^8^	**1.43 × 10^6^**	**2.45 × 10^5^**	**1.02 × 10^6^**	3.06 × 10^7^	4.20 × 10^6^	2.41 × 10^7^
f3	3.57 × 10^10^	2.32 × 10^10^	1.25 × 10^10^	**1.54 × 10^7^**	**1.66 × 10^7^**	**8.51 × 10^5^**	1.22 × 10^9^	3.84 × 10^8^	5.98 × 10^8^
f4	5.44 × 10^4^	7.80 × 10^3^	3.61 × 10^4^	3.11 × 10^4^	3.86 × 10^3^	2.25 × 10^4^	**1.37 × 10^4^**	**2.19 × 10^3^**	**9.78 × 10^3^**
f5	3.87 × 10^3^	7.35 × 10^2^	1.51 × 10^3^	**2.02 × 10^−3^**	**3.04 × 10^−4^**	**1.42 × 10^−3^**	1.75 × 10^2^	9.78 × 10^1^	1.21 × 10^2^
f6	5.23 × 10^2^	6.53 × 10^1^	3.25 × 10^2^	**2.33 × 10^1^**	2.14 × 10^1^	**4.41 × 10^−1^**	9.90 × 10^1^	**1.81 × 10^1^**	5.57 × 10^1^
f7	1.62 × 10^2^	4.90 × 10^1^	1.15 × 10^2^	**1.41 × 10^1^**	9.58 × 10^0^	**2.49 × 10^0^**	4.57 × 10^1^	**8.71 × 10^0^**	3.15 × 10^1^
f8	2.10 × 10^1^	4.54 × 10^−2^	**2.08 × 10^1^**	2.10 × 10^1^	4.77 × 10^−2^	**2.08 × 10^1^**	**2.09 × 10^1^**	**5.13 × 10^−2^**	**2.08 × 10^1^**
f9	**3.92 × 10^1^**	**1.01 × 10^0^**	3.65 × 10^1^	3.96 × 10^1^	1.09 × 10^0^	**3.53 × 10^1^**	3.94 × 10^1^	1.44 × 10^0^	3.63 × 10^1^
f10	9.29 × 10^2^	9.79 × 10^1^	7.07 × 10^2^	**2.45 × 10^−1^**	**1.89 × 10^−1^**	**6.16 × 10^−2^**	5.83 × 10^1^	6.90 × 10^0^	4.24 × 10^1^
f11	3.09 × 10^2^	2.03 × 10^1^	2.67 × 10^2^	**1.66 × 10^2^**	5.32 × 10^1^	**6.96 × 10^0^**	2.14 × 10^2^	**1.36 × 10^1^**	1.83 × 10^2^
f12	3.15 × 10^2^	1.31 × 10^1^	2.86 × 10^2^	**1.56 × 10^2^**	5.47 × 10^1^	**2.19 × 10^1^**	2.12 × 10^2^	**1.28 × 10^1^**	1.82 × 10^2^
f13	3.12 × 10^2^	1.61 × 10^1^	2.66 × 10^2^	**1.84 × 10^2^**	1.95 × 10^1^	**1.07 × 10^2^**	2.16 × 10^2^	**1.40 × 10^1^**	1.73 × 10^2^
f14	7.40 × 10^3^	2.46 × 10^2^	6.90 × 10^3^	**7.29 × 10^3^**	3.02 × 10^2^	**6.56 × 10^3^**	7.37 × 10^3^	**2.20 × 10^2^**	6.89 × 10^3^
f15	7.38 × 10^3^	2.89 × 10^2^	6.51 × 10^3^	7.35 × 10^3^	**2.82 × 10^2^**	6.63 × 10^3^	**7.27 × 10^3^**	3.12 × 10^2^	**6.31 × 10^3^**
f16	2.51 × 10^0^	2.68 × 10^−1^	1.91 × 10^0^	2.53 × 10^0^	**2.92 × 10^−1^**	**1.77 × 10^0^**	**2.40 × 10^0^**	2.85 × 10^−1^	1.85 × 10^0^
f17	3.48 × 10^2^	**1.21 × 10^1^**	3.19 × 10^2^	**2.10 × 10^2^**	1.24 × 10^1^	**1.79 × 10^2^**	2.39 × 10^2^	**1.21 × 10^1^**	1.99 × 10^2^
f18	3.49 × $10^{2}$	2.61 × $10^{1}$	3.12 × $10^{2}$	**2.12 × $10^{2}$**	1.18 × $10^{1}$	**1.88 × $10^{2}$**	2.41 × $10^{2}$	**9.65 × $10^{0}$**	2.25 × $10^{2}$
f19	3.08 × $10^{2}$	2.43 × $10^{2}$	3.75 × $10^{1}$	**1.52 × $10^{1}$**	1.40 × $10^{0}$	**1.13 × $10^{1}$**	2.02 × $10^{1}$	**1.29 × $10^{0}$**	1.76 × $10^{1}$
f20	1.45 × $10^{1}$	2.25 × $10^{-1}$	1.39 × $10^{1}$	**1.19 × $10^{1}$**	2.69 × $10^{-1}$	**1.12 × $10^{1}$**	1.26 × $10^{1}$	**4.78 × $10^{-1}$**	1.17 × $10^{1}$
f21	1.25 × $10^{3}$	**9.05 × $10^{1}$**	1.04 × $10^{3}$	**3.46 × $10^{2}$**	9.09 × $10^{1}$	**2.00 × $10^{2}$**	4.12 × $10^{2}$	9.46 × $10^{1}$	3.39 × $10^{2}$
f22	7.74 × $10^{3}$	**3.07 × $10^{2}$**	6.85 × $10^{3}$	**7.65 × $10^{3}$**	3.39 × $10^{2}$	6.80 × $10^{3}$	7.67 × $10^{3}$	3.44 × $10^{2}$	**6.74 × $10^{3}$**
f23	7.91 × $10^{3}$	**2.80 × $10^{2}$**	7.10 × $10^{3}$	**7.76 × $10^{3}$**	3.24 × $10^{2}$	**7.09 × $10^{3}$**	7.82 × $10^{3}$	3.16 × $10^{2}$	7.10 × $10^{3}$
f24	3.15 × $10^{2}$	1.13 × $10^{1}$	2.96 × $10^{2}$	**2.30 × $10^{2}$**	6.72 × $10^{0}$	**2.16 × $10^{2}$**	2.45 × $10^{2}$	**2.51 × $10^{0}$**	2.37 × $10^{2}$
f25	3.47 × $10^{2}$	**5.10 × $10^{0}$**	3.31 × $10^{2}$	**2.89 × $10^{2}$**	2.81 × $10^{1}$	2.51 × $10^{2}$	2.92 × $10^{2}$	4.08 × $10^{1}$	**2.35 × $10^{2}$**
f26	2.24 × $10^{2}$	**5.86 × $10^{0}$**	2.11 × $10^{2}$	**2.10 × $10^{2}$**	3.08 × $10^{1}$	**2.00 × $10^{2}$**	2.22 × $10^{2}$	5.02 × $10^{1}$	2.01 × $10^{2}$
f27	1.40 × $10^{3}$	**2.94 × $10^{1}$**	1.32 × $10^{3}$	**6.58 × $10^{2}$**	7.29 × $10^{1}$	**5.12 × $10^{2}$**	7.92 × $10^{2}$	3.58 × $10^{1}$	6.96 × $10^{2}$
f28	2.19 × $10^{3}$	2.10 × $10^{2}$	1.00 × $10^{3}$	**3.00 × $10^{2}$**	**1.29 × $10^{-13}$**	**3.00 × $10^{2}$**	4.28 × $10^{2}$	3.64 × $10^{1}$	3.75 × $10^{2}$

**Table 5:** Wilcoxon sign rank test results for 51 repeated experiments at 30 dimensions, the bold part indicating the cases where the test was found to be true.

*p*-Value		Crouping	BIP-Mean vs. BIP	MQHOA-wmn vs. BIP	BIP-Mean vs. MQHOA-wmn
f(x)					
f1			**9.49 × $10^{-19}$**	**9.7555 × $10^{-10}$**	**1.46343 × $10^{-11}$**
f2			**3.30 × $10^{-18}$**	**3.01986 × $10^{-11}$**	**3.01986 × $10^{-11}$**
f3			**3.30 × $10^{-18}$**	**3.01986 × $10^{-11}$**	**3.01986 × $10^{-11}$**
f4			**4.70 × $10^{-18}$**	**3.01986 × $10^{-11}$**	**3.01986 × $10^{-11}$**
f5			**3.30 × $10^{-18}$**	**3.01986 × $10^{-11}$**	**3.01986 × $10^{-11}$**
f6			**3.30 × $10^{-18}$**	**3.01986 × $10^{-11}$**	**1.20567 × $10^{-10}$**
f7			**3.30 × $10^{-18}$**	**3.01986 × $10^{-11}$**	**1.77691 × $10^{-10}$**
f8			7.13 × $10^{-1}$	0.122352926	0.923442132
f9			**1.75 × $10^{-2}$**	0.482516904	0.325526587
f10			**3.30 × $10^{-18}$**	**3.01986 × $10^{-11}$**	**3.01608 × $10^{-11}$**
f11			**3.30 × $10^{-18}$**	**3.01986 × $10^{-11}$**	**7.08811 × $10^{-8}$**
f12			**3.30 × $10^{-18}$**	**3.01986 × $10^{-11}$**	**2.0338 × $10^{-9}$**
f13			**3.30 × $10^{-18}$**	**3.01986 × $10^{-11}$**	**1.84999 × $10^{-8}$**
f14			1.41 × $10^{-1}$	0.911708975	0.363222313
f15			5.12 × $10^{-1}$	0.067868861	0.982307053
f16			6.54 × $10^{-1}$	0.200948897	0.059427915
f17			**3.30 × $10^{-18}$**	**3.01986 × $10^{-11}$**	**4.1825 × $10^{-9}$**
f18			**3.30 × $10^{-18}$**	**3.01986 × $10^{-11}$**	**4.97517 × $10^{-11}$**
f19			**3.30 × $10^{-18}$**	**3.01986 × $10^{-11}$**	**3.01986 × $10^{-11}$**
f20			**3.30 × $10^{-18}$**	**3.01986 × $10^{-11}$**	**3.96477 × $10^{-8}$**
f21			**4.09 × $10^{-19}$**	**3.01986 × $10^{-11}$**	**9.45134 × $10^{-5}$**
f22			1.43 × $10^{-1}$	0.311187643	0.853381737
f23			**1.63 × $10^{-2}$**	0.728265296	0.233988916
f24			**3.30 × $10^{-18}$**	**3.01986 × $10^{-11}$**	**4.07716 × $10^{-11}$**
f25			**3.30 × $10^{-18}$**	**1.95678 × $10^{-10}$**	0.970516051
f26			**1.63 × $10^{-14}$**	**1.10772 × $10^{-6}$**	0.371077032
f27			**3.30 × $10^{-18}$**	**3.01986 × $10^{-11}$**	**5.96731 × $10^{-9}$**
f28			**1.39 × $10^{-20}$**	**3.01986 × $10^{-11}$**	**1.21178 × $10^{-12}$**

## Data Availability

The raw data supporting the conclusions of this article will be made available by the authors on request.

## Abbreviations

The following abbreviations are used in this manuscript:

SI	Swarm Intelligence
PSO	Particle Swarm Optimization
QPSO	Quantum-behaved Particle swarm Optimization
WQPSO	Weighted Quantum-behaved Particle swarm Optimization
MPSO	Median-oriented Particle Swarm Optimization
MGWO	Mean Gray Wolf Optimization
QMBA	Quantum-behaved Bat Algorithm with Mean Best Position Directed
EHO	Elephant Herding Optimization
CS	Cuckoo Search
GQBA	Gaussian Quantum Bat Algorithm
SHADE	Success-History Based Parameter Adaptation Differential Evolution
CSHADE	Center-based mutation for Success-History based parameter Adaptation
	Differential Evolution
MQHOA	Multi-scale quantum harmonic optimization algorithm
QDF	Quantum Dynamic Frame
QDE	Quantum Dynamic Equation
BIP	Basic Iteration Process
DWF	Double Well Function
MQHOA	Multi-scale Quantum Harmonic Optimization Algorithm
TELA	Two Energy Level Approximation
